# Impact of Diabetes Mellitus on the Outcome of Pancreatic Cancer

**DOI:** 10.1371/journal.pone.0098511

**Published:** 2014-05-30

**Authors:** Muhammad Shaalan Beg, Alok Kumar Dwivedi, Syed Arif Ahmad, Sadia Ali, Olugbenga Olowokure

**Affiliations:** 1 Department of Internal Medicine, Division of Hematology/Oncology, University of Texas Southwestern Medical Center, Dallas, Texas, United States of America; 2 Division of Biostatistics and Epidemiology, Department of Biomedical Sciences, Texas Tech University Health Sciences Center, El Paso, Texas, United States of America; 3 Department of Surgery, University of Cincinnati College of Medicine, Cincinnati, Ohio, United States of America; 4 Department of Internal Medicine, Division of Endocrinology, University of Texas Southwestern Medical Center, Dallas, Texas, United States of America; 5 Division of Hematology/Oncology, University of Cincinnati College of Medicine, Cincinnati, Ohio, United States of America; Spanish National Cancer Centre (CNIO), Spain

## Abstract

**Introduction:**

Diabetes mellitus (DM) has the potential to impact the pathogenesis, treatment, and outcome of pancreatic cancer. This study evaluates the impact of DM on pancreatic cancer survival.

**Methods:**

We conducted a retrospective cohort study from the Veterans Affairs (VA) Central Cancer Registry (VACCR) for pancreatic cancer cases between 1995 and 2008. DM and no-DM cases were identified from comorbidity data. Univariate and multivariable analysis was performed. Multiple imputation method was employed to account for missing variables.

**Results:**

Of 8,466 cases of pancreatic cancer DM status was known in 4728 cases that comprised this analysis. Males accounted for 97.7% cases, and 78% were white. Overall survival was 4.2 months in DM group and 3.6 months in the no-DM group. In multivariable analysis, DM had a HR = 0.91 (0.849–0.974). This finding persisted after accounting for missing variables using multiple imputations method with the HR in DM group of 0.93 (0.867–0.997).

**Conclusions:**

Our data suggest DM is associated with a reduction in risk of death in pancreatic cancer. Future studies should be directed towards examining this association, specifically impact of DM medications on cancer outcome.

## Introduction

Pancreatic cancer is a cause of significant morbidity and mortality and effective treatment strategies are lacking. In 2012 there were an estimated 43,920 new cases and 37,390 deaths from pancreas cancer in the United States [1]. The 5-year overall survival of patients with pancreatic cancer is less than 5% which makes pancreatic cancer the fourth leading cause of cancer-related mortality in North America [2]. Diabetes mellitus (DM) occurs in up to 68% of patients with pancreatic cancer with 40% developing DM in the 36 months preceding their cancer diagnosis [3]. DM is also a recognized risk factor for the development of pancreatic cancer [4].

The development of DM in patients with pancreatic cancer is likely secondary to a combination of factors leading to a marked decline in pancreatic β cell function and profound peripheral insulin resistance. Patients with advanced PC display many of the metabolic abnormalities seen in type 2 DM including glucose intolerance, increased hepatic glucose production and insulin resistance [3], [4]. Insulin resistance can lead to increased muscle proteolysis and contribute to inhibition of protein anabolism which can result in cachexia, a syndrome characterized by significant loss of adipose and skeletal muscle tissue [5], [6]. Pancreatic cancer patients with cachexia are less likely to undergo curative surgery and have increased postoperative mortality [4], [7]. However, there is an increasingly recognized interaction of commonly used DM medications as having an anticancer role which may further influence the outcome of pancreatic cancer [8]. Therefore, the etiology of diabetes, impact of insulin resistance related cachexia, as well as DM treatment, has the potential to impact pancreatic cancer outcome.

## Methods

### Ethics Statement

This study is a retrospective analysis of an existing cancer registry. Deidentified data was available for download from the VA Intranet. Data was accessed January 2010. This study involved study of data from an existing source which waspublicly available and information was recorded in a manner that subjects could not be identified. This study qualifies as exempt from IRB review in accordance with NIH Office of Subject Research (45 CFR 46.101(b)(4)). This study was determined to be ‘exempt’ from Institutional Review Board (IRB) review by the UT Southwestern Medical Center IRB.

We conducted a retrospective cohort study to investigate whether overall survival (OS) was different in pancreatic cancer cases with DM as compared to those with no-DM. Data was extracted from the Veterans Affairs Central Cancer Registry (VACCR), which aggregates cancer data from Veterans Affairs (VA) centers across the United States. The reference date for the VACCR is January 1, 1995. PC cases from the VACCR between 1995 and 2008 were included and the DM status, along with demographic, cancer pathology and treatment, life style information were collected. Any unknown DM status cases were excluded from the study.

The primary outcome, overall survival (OS), was measured from the date of diagnosis till date of death or date of last follow up (censored). The main exposure variable in this study was known presence or absence of DM. A total of 11 cofactors (DM, age, gender, race, alcohol, tobacco, primary site, stage, surgery, chemotherapy and radiation therapy) were considered in the analysis. Age at diagnosis was presented using mean and standard deviation. The median duration of follow up was estimated and reported along with the range. The categorical variables were reported using frequencies and proportions. To assess the effect of cofactors on OS, univariate and multivariable Cox proportional hazards models were developed. The results of univariate Cox models were reported using unadjusted hazard ratio (UHR) with their 95% confidence intervals (CI) whereas adjusted hazard ratio (AHR) and their 95% CIs were used to report the effect of cofactors in multivariable Cox model.

To accommodate for missing data multiple imputation (MI) was used using the Markov Chain Monte Carlo (MCMC) method [9]–[11]. Imputed values are rounded and bounded during imputation so that imputed values match the format of observed values as well as existing ranges. MI relies on the assumption that missing values depend on other variables in the dataset i.e., missing at random (MAR). To determine if missing data was MAR we evaluated Tetrachoric Correlation and Pearson correlation between missing variables and other variables included in the multivariable model. No significant correlation was found for a majority of variable supporting that missing data was missing at random. MI procedure estimates n (here, we considered n = 5) complete data sets. Each data set was analyzed using the Cox regression. Finally, the results from each of the Cox models were pooled to compute the model estimates. To assess the effect of multiple imputations, we compared the results of the Cox model developed on excluding all missing information as well as by incorporating ‘missing’ as a level in the considered variable. Kaplan Meier curve was constructed for OS. The p-values less than 5% were regarded as significant results. All the statistical analyses were conducted using SAS 9.2.

## Results

A total of 8466 pancreatic cancer cases were identified from the VACCR. DM status was known in 4728 cases. Thus, a total of 3738 cases were excluded from the analysis. Mean age was 67.2 years, 97.7% cases were male and 51.9% cancers originated from the head of the pancreas. Stage IV disease was present in 46.5% cases (Table 1).

**Table 1:** Baseline characteristics (n = 4728).

Variables	Total (%)
Age (years)	67.2
Diabetes	
Yes	1326 (28.)
No	3402 (72.0)
Gender	
Male	4617 (97.7)
Female	111 (2.3)
Race	
White	3689 (78.0)
Non-White	1039 (22.0)
Site	
Head of pancreas	2452 (51.9)
Body of pancreas	478 (10.1)
Tail of pancreas	573 (12.1)
Other	323 (6.8)
Unknown	902 (19.1)
Stage	
Stage 1–3	564 (11.9)
Stage 4	2198 (46.5)
Other stage/unknown	1966 (41.6)

Of 4728, 70 cases had missing follow up data. Survival analysis was performed on the remaining 4658 cases. Median follow up was 3.6 (interquartile range: 1.3–7.4) months. The median OS was 3.8 in the study group. DM patients had a longer median OS as compared to non-DM patients (4.2 vs. 3.6 months, p = 0.04) (Table 2). In unadjusted analysis, all variables including DM were found to be associated with OS except race and alcohol (Table 3). In multivariable analysis, DM, age, tobacco use, disease site, stage, chemotherapy and surgery were associated with OS while race, alcohol and radiation therapy were not found to be associated (Table 3). DM patients had 9% lower risk of death as compared to non-DM patients (AHR: 0.91, p = 0.006) after controlling for other significant variables (Table 3). A statistically significant difference of DM status was not detected after excluding all missing variable information (Table 4). Lastly, multiple imputations were carried out for all missing cofactor data (Table 4). After multiple imputation of missing observations DM patients had a lower risk of death than non-DM after controlling other cofactors in the study [HR: 0.93, p = 0.0397]. Therefore findings in the multivariable analysis were consistent after MI for missing data.

**Table 2:** Overall survival of pancreatic cancer (PC) cases according to DM status (N = 4658).

	N	Median survival (months)	95% Confidence Interval
All PC cases	4658	3.8	3.6–4.0
with DM	1312	4.2	3.9–4.7
Non-DM	3346	3.6	3.3–3.8

**Table 3:** Unadjusted and adjusted association of variables with mortality.

Variables	Unadjusted Analysis (n = 4658*)			Adjusted Analysis (n = 4658*)		
	HR	95% CI	p-value	AHR	95% CI	p-value
DM						
Yes	0.934	0.873–1	0.0491	0.909	0.849–0.974	0.0065
No(referent)						
Age	1.018	1.015–1.021	<.0001	1.012	1.009–1.015	<.0001
Gender						
Female	0.763	0.619–0.941	0.0115	0.827	0.669–1.023	0.0797
Male(referent)						
Race						
Non-White	1.018	0.946–1.096	0.6297	1.053	0.977–1.134	0.1753
White(referent)						
Alcohol						
Current use	0.939	0.866–1.017	0.1236	0.927	0.85–1.011	0.0858
Previous use	0.979	0.901–1.064	0.6164	1.008	0.922–1.103	0.8549
Unknown	1.048	0.952–1.155	0.34	0.925	0.797–1.074	0.3057
No (referent)						
Tobacco						
Current use	1.036	0.948–1.132	0.4347	1.171	1.062–1.29	0.0015
Previous use	1.056	0.966–1.154	0.2333	1.077	0.979–1.185	0.1276
Unknown	1.173	1.051–1.309	0.0043	1.196	1.013–1.412	0.0343
No(referent)						
Site						
Body of pancreas	1.36	1.227–1.508	<.0001	1.145	1.032–1.27	0.0108
Tail of pancreas	1.22	1.106–1.346	<.0001	1.02	0.922–1.129	0.6957
Other	1.224	1.082–1.386	0.0013	1.125	0.993–1.273	0.0639
Unknown	1.435	1.323–1.556	<.0001	1.201	1.106–1.303	<.0001
head(referent)						
Stage						
Stage 4	2.259	2.045–2.497	<.0001	2.046	1.846–2.269	<.0001
Other stage/unknown	1.04	0.94–1.15	0.451	1.088	0.981–1.207	0.1114
Stage1–3(referent)						
Chemo						
Yes	0.517	0.484–0.551	<.0001	0.506	0.472–0.544	<.0001
No(referent)						
Surgery						
Yes	0.335	0.304–0.369	<.0001	0.423	0.381–0.469	<.0001
No(referent)						
Radiation						
Yes	0.484	0.44–0.533	<.0001	0.925	0.835–1.024	0.134
No(referent)						

*70 records were excluded due to missing follow up.

**Table 4:** Adjusted association of DM with and without missing imputation.

Variables	After excluding all missing (n = 1815)			After multiple imputation (n = 4728)		
	AHR	95% CI	p-value	AHR	95% CI	p-value
DM						
Yes	0.981	0.883–1.09	0.7199	0.93	0.867–0.997	0.0397
No(referent)						
Age	1.011	1.006–1.016	<.0001	1.01	1.007–1.014	<.0001
Gender						
Female	0.795	0.582–1.086	0.1501	0.834	0.66–1.055	0.1298
Male(referent)						
Race						
Non-White	1.212	1.078–1.363	0.0013	0.956	0.886–1.032	0.2473
White(referent)						
Alcohol						
Current use	0.922	0.812–1.047	0.2104	1.145	1.037–1.263	0.0074
Previous use	1.045	0.914–1.194	0.5197	1.072	0.957–1.201	0.223
No (referent)						
Tobacco						
Current use	1.16	1.006–1.338	0.0409	1.149	1.007–1.312	0.04
Previous use	0.968	0.84–1.114	0.649	0.974	0.895–1.06	0.5458
No(referent)						
Site						
Body of pancreas	1.319	1.153–1.509	<.0001	1.025	0.946–1.111	0.5479
Tail of pancreas	1.141	0.999–1.302	0.0514	1.14	1.024–1.269	0.0185
Other	1.222	1.038–1.44	0.0162	1.071	0.953–1.203	0.2371
head(referent)						
Stage						
Stage 4	2.045	1.801–2.322	<.0001	1.756	1.609–1.915	<.0001
Stage1–3(referent)						
Chemo						
Yes	0.432	0.389–0.481	<.0001	0.563	0.524–0.605	<.0001
No(referent)						
Surgery						
Yes	0.436	0.329–0.577	<.0001	0.411	0.371–0.456	<.0001
No(referent)						
Radiation						
Yes	0.968	0.819–1.146	0.708	0.934	0.84–1.04	0.2116
No(referent)						

## Discussion

Our analysis from a large national database suggests that DM is associated with an improvement in pancreatic cancer outcome. The effect of DM status on OS was modest and found to be consistent in the adjusted multivariable analysis with or without MI for missing data. Existing literature on this association largely stems from smaller cohorts and results have been conflicting. Reports have suggested that DM has been associated with worse outcome in patients with pancreatic adenocarcinoma [12]–[15]. Various etiologies have been proposed for increased mortality in pancreatic cancer patients with DM. These include treatment with exogenous insulin, increased bioavailability of insulin-like growth factor-1 (IGF-1) combined with increased IGF-1 receptors in pancreatic cancer [16]–[18]. One of the largest studies to demonstrate DM was associated with increased PC mortality arises from the Cancer Prevention Study II (CPS-II) a prospective mortality study of 1.2 million Americans [19]. Whereas the CPS-II is a large, prospective study, investigators relied on patient self-reported information on a baseline survey which was performed in 1982. As cause of death was ascertained through the National Death index in 1998, this study may have been unable to identify change in DM status among enrollees during that time interval. As a large proportion of pancreatic cancer patients develop DM within two years of cancer diagnosis, a significant portion of DM cases were likely missed. A more accurate assessment of DM status would be the presence of DM at the time of pancreatic cancer diagnosis. In addition, with the advent of new DM medications, the management of DM has improved dramatically over the past few decades affecting the survival of diabetics in general. Another single institution study suggested DM along with hypertension, cardiovascular disease and chronic obstructive pulmonary disease predicted patients at high risk of early mortality from pancreatic resection, but the impact of this risk score in patients with metastatic disease was not assessed [20].These findings of poor prognosis of pancreatic cancer in DM have not been confirmed in other studies that have shown DM patients have similar presentation, stage, survival and postoperative complications than non-DM patients [21]–[23]. Our analysis is of a large, national database of VA centers. The VA system has the benefit of being a single payer system, where socio-demographic factors, access to health care and insurance coverage may not affect medical care delivery, and therefore allow for better comparison of the effect of DM on pancreatic cancer outcome.

The mechanism behind the close association of DM and pancreatic cancer is not entirely clear, but has the potential to offer a therapeutic opportunity. This mechanism may not be a result of pancreatic beta-cell replacement by cancer cells as initially believed, but rather a result of cancer cells inducing a state of peripheral insulin resistance. The later concept is supported by the fact that DM has been shown to improve after PC resection [24]. Also, other factors may be at play, such as insulin and beta cell autoantibodies which have been attributed to the high incidence of DM in pancreatic cancer [25]. Multiple studies have reported an association of DM and increased risk of pancreatic cancer, compared with non-DM population. Two meta-analyses have suggested the relative risk of pancreatic cancer is inversely associated with the duration of DM with the highest risk of pancreatic cancer found in patients diagnosed with DM for less than 1 year [26], [27].

Sadeghi et al evaluated the impact of DM treatment on the outcome of pancreatic cancer from a single institution and suggested a role of metformin on improving the outcome of diabetics with pancreatic cancer [8]. This potential antineoplastic effect of metformin has also been seen in response to neoadjuvant therapy in breast cancer with a higher pathologic complete response rate in metformin [28]. The phenomenon of improvement in cancer outcome appears specific to metformin and is not seen with insulin or sulfonylureas [29]. Metformin use has also been associated with reduced risk of pancreatic cancer, a phenomenon not seen with insulin or secretagogues [30]. Medication information is not readily available from cancer registries, including the VACCR, and as such was not available for our analysis. However, one possible explanation of the improved outcome in the DM group may reflect metformin effect. Unlike other larger cancer data sources, patients in the VACCR have uniform access to health care due to the nature of the one payer VA medical system. As such, a larger proportion of patients are more likely to be on appropriate DM therapy, which typically includes metformin as a front line agent, and may abrogate the potential adverse effect of insulin resistance or IGF-1 on survival and account for our improved survival findings.

One other explanation behind conflicting results of DM on pancreatic cancer prognosis may be differing pathophysiological mechanisms in cases that have long standing DM compared to those who develop DM close to their pancreatic cancer diagnosis. In a sample of resected pancreatic cancer cases, Ben et al showed that although those who developed DM within 2 years of pancreatic cancer diagnosis had poor prognostic features on pathology and worse survival, but the prognosis of long standing DM patients was not worse when compared with non-DM patients [31]. Database analyses such as ours are inherently unable to differentiate these two groups of DM cases which likely represent two different patient populations.

Limitations of our study include issues pertaining to most large database studies. We analyzed comorbid illness coding in the VACCR which records up to six co-morbid conditions for each case. Whereas the availability of comorbid conditions in the VACCR is a major strength of our study and permitted this analysis, various aspects of DM history such as duration, occurrence after pancreatic cancer diagnosis, and type of DM treatments (biguanides, sulfonylureas, insulin etc) were not known. In addition, other comorbid conditions including hypertension, coronary artery disease, dementia, and previous cancer diagnosis were not individually assessed in this analysis. Each of these factors may independently affect outcome of pancreatic cancer and warrants further evaluation, ideally in a larger prospective study with special focus on DM medications and interplay of comorbid conditions. The effect of DM status on OS did not reach statistical significance level after excluding missing covariate information. This is explainable by reduced power to detect significant differences in survival between DM and non-DM patients after excluding all missing data. The multiple imputations methods employed in our analysis is a rigorous and valid strategy for dealing with missing values. Each missing values are replaced with plausible values that represent the uncertainty about the right value to impute and preserve power.

Our analysis of a large single-payer national database suggests improved survival and reduced risk of death in the DM population. Whereas the reasons behind these findings are not entirely clear from this analysis, these findings may be a function of high rates of metformin use among DM patients in the VA system [32]. Also, patients in the VA system have uniform access to healthcare, therefore socio-demographic factors may play less of a role in determining disease management and may explain our findings. Despite the above mentioned limitations which affect most database analyses, these findings are intriguing, and need to be confirmed in a large, preferably prospective, study with detailed analysis of DM medications and in the context of other comorbidities. Greater understanding of the molecular mechanisms at play in oncogenesis, such as the mammalian target of rapamycin (mTOR) pathway, has shown that the interplay between DM and cancer is much more complex than originally thought [33]. Therefore greater understanding of the interaction of DM and pancreatic cancer is essential to apply these advances in the clinic.
